# Role of endothelial microRNA 155 on capillary leakage in systemic inflammation

**DOI:** 10.1186/s13054-021-03500-0

**Published:** 2021-02-22

**Authors:** Valerie Etzrodt, Temitayo O. Idowu, Heiko Schenk, Benjamin Seeliger, Antje Prasse, Kristina Thamm, Thorben Pape, Janina Müller-Deile, Matijs van Meurs, Thomas Thum, Ankita Garg, Robert Geffers, Klaus Stahl, Samir M. Parikh, Hermann Haller, Sascha David

**Affiliations:** 1 Division of Nephrology and Hypertension, Hannover Medical School, Hannover, Germany; 2 Department of Respiratory Medicine and German Center for Lung Research, Hannover Medical School, Hannover, Germany; 3 Division of Nephrology and Hypertension, University Hospital Erlangen, Erlangen, Germany; 4 Department of Critical Care, University of Groningen, University Medical Center Groningen, Groningen, The Netherlands; 5 Institute of Molecular and Translational Therapeutic Strategies, Hannover Medical School, Hannover, Germany; 6 Helmholtz Centre for Infection Research Brunswick, Brunswick, Germany; 7 Division of Gastroenterology, Hepatology and Endocrinology, Hannover Medical School, Hannover, Germany; 8 Center for Vascular Biology Research, Beth Israel Deaconess Medical Center and Harvard Medical School, Boston, MA USA; 9 Mount Desert Island Biological Laboratory, Bar Harbor, ME USA; 10 Institute of Intensive Care Medicine, University Hospital Zurich, Rämistrasse 100, 8092 Zurich, Switzerland

**Keywords:** Tight junctions, Respiratory distress syndrome, MicroRNAs, Endothelium, Sepsis

## Abstract

**Background:**

Capillary leakage is a key contributor to the pathological host response to infections. The underlying mechanisms remain incompletely understood, and the role of microRNAs (MIR) has not been investigated in detail. We hypothesized that specific MIRs might be regulated directly in the endothelium thereby contributing to vascular leakage.

**Methods:**

SmallRNA sequencing of endotoxemic murine pulmonary endothelial cells (ECs) was done to detect regulated vascular MIRs. In vivo models: transgenic zebrafish (flk1:mCherry/l-fabp:eGFP-DPB), knockout/wildtype mouse (B6.Cg-Mir155tm1.1Rsky/J); disease models: LPS 17.5 mg/kgBW and cecal ligation and puncture (CLP); in vitro models: stimulated human umbilical vein EC (HUVECs), transendothelial electrical resistance.

**Results:**

Endothelial MIR155 was identified as a promising candidate in endotoxemic murine pulmonary ECs (25 × upregulation). Experimental overexpression in a transgenic zebrafish line and in HUVECs was sufficient to induce spontaneous vascular leakage. To the contrary, genetic MIR155 reduction protects against permeability both in vitro and in endotoxemia in vivo in MIR155 heterozygote knockout mice thereby improving survival by 40%. A tight junction protein, Claudin-1, was down-regulated both in endotoxemia and by experimental MIR155 overexpression. Translationally, MIR155 was detectable at high levels in bronchoalveolar fluid of patients with ARDS compared to healthy human subjects.

**Conclusions:**

We found that MIR155 is upregulated in the endothelium in mouse and men as part of a systemic inflammatory response and might contribute to the pathophysiology of vascular leakage in a Claudin-1-dependent manner. Future studies have to clarify whether MIR155 could be a potential therapeutic target.

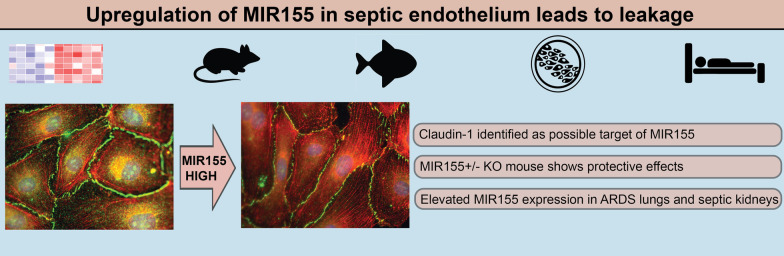

## Background

Sepsis is defined as a life-threatening pathological host response to infection [1]. Key components of this host response are (1) a dysfunctional (often overwhelming) immune reaction, (2) intravascular coagulopathy and (3) global endothelial dysfunction all together leading to a microvascular stasis, organ hypo-perfusion and ultimately fatal multi-organ failure. The mortality ranges between 32 and 56% [1, 2]. A deleterious vascular maladaptation can occur in virtually all syndromes that are characterized by a systemic inflammatory response both in septic and aseptic conditions (e.g., pancreatitis, burns, post-cardiopulmonary bypass) [3]. The underlying molecular mechanisms have been investigated for some time but are still incompletely understood. The molecular basis for maintenance of cell–cell contacts is the so-called *adherens* and *tight junctions*, transmembrane proteins that dynamically link adjacent cells to one another [4]. These junctions consist of a variety of proteins that are connected and form a robust scaffold with the intracellular actin cytoskeleton. Targeting one component of these complicated structures might have catastrophic effects on the stability of the whole complex. Some of these junctional proteins have been demonstrated to be altered in expression, structure and/or localization in systemic inflammation (e.g., VE-cadherin, catenins, Claudin-1 [5]).

MicroRNAs (MIR)—small non-coding RNAs that have the ability to simultaneously regulate a variety of proteins—have not been investigated in detail in modulating the endothelial junctional apparatus thus controlling permeability [6]. In this context, one could hypothesize, that MIRs could trigger mechanisms that affect endothelial barrier function by targeting both essential proteins of the junctional apparatus or of the cytoskeleton either in a beneficial or harmful nature.

We hypothesized that specific MIRs might be up-/or down-regulated in systemic inflamed endothelium thereby contributing to the degradation of critical components of endothelial junctions or cytoskeletal components. To analyze this, we first performed an unbiased MIR analysis using a small RNA sequencing strategy from endotoxemic murine pulmonary endothelial cells and identified MIR155 as a potential candidate of interest. We then analyzed endothelial MIR155 regulation in systemic inflammation in transgenic zebrafish, wildtype and knockout mice, human endothelial cells, septic human kidney biopsies, and in bronchoalveolar lavage fluid (BALf) and serum samples from ARDS patients.

## Material and methods

We followed the ARRIVE guidelines (www.arriveguidelines.org) throughout the investigation whenever applicable.

### Antibodies and reagents

All chemical and reagents, unless otherwise specified, were purchased from Sigma-Aldrich. Antibodies against Claudin-1, ZO-1, GAPDH and Alexa Fluor 546 Phalloidin, 4′,6-diamidino-2-phenylindole (DAPI) (Sigma-Aldrich, St.Louis, MO) and Aqua-Poly/Mount were used.

### miRNA analysis using smallRNA-Seq

RNA sequencing library was generated from 0.1 to 1 µg of total RNA using TruSeq® Small RNA Library Prep Kits v2 (Illumina) according to manufacturer’s protocols. The libraries were sequenced on Illumina HiSeq2500 using TruSeq SBS Kit v3-HS (50 cycles, single ended run) with an average of 10 × 106 reads per RNA sample. FASTQ files were trimmed with fastq-mcf (ea-utils, https://expressionanalysis.github.io/ea-utils/) removing Illumina RNA adapter sequences (TGGAATTCTCGGGTGCCAAGG) and nucleotides with phred scores below 20. [7–10]

### Cell culture studies

Human umbilical vein endothelial cells (HUVECs) were isolated from human umbilical veins (donor approval, Hannover Medical School Nr. 1303-2012). Specific miRCURY LNA miRNA mimic (No. 339173) (Qiagen, Hilden, Germany) was used to overexpress the microRNA 155. To inhibit the microRNA 155, the miRCURY LNA miRNA Inhibitor 5 nM was used (No. 339121) (Qiagen, Hilden, Germany).

### Mouse studies

All mouse experiments were approved by the local authorities at Hannover Medical School and conducted in accordance with institutional and governmental guidelines (LAVES Lower Saxony, Ref. No. 18/2817). Male B6.Cg-Mir155tm1.1Rsky/J were used as MIR155 knockout mouse. The mice, 10–12 weeks of age, were either injected intraperitoneally (i.p.) with 17.5 mg/kg bodyweight lipopolysaccharide (LPS) or a cecal ligature puncture (CLP) as described elsewhere [11] was conducted. Briefly, the LPS originated from Escherichia coli, serotype O111:B4 (Sigma-Aldrich, St. Louis, MO) dissolved in 10 mL/kg sterile NaCl. The injected mice were monitored and scored according to Additional file 1: Table S4. After 16 h, they were killed and organs harvested. For the performance of the CLP experiment, the mice were anaesthetized with isoflurane (1–2% in medical air) and a midline laparotomy was placed by a single-blinded operator. The anti-mesenteric border was ligated, and through one single 20G needle puncture 1 mm of stool was extracted. As a sham surgery, laparotomy with cecal mobilization was performed. Followed by the two-layering closure, mice were given 200 µL of NaCl fluids s.c. and for analgesia once 10 mg/mL Butorphanol (Zoetis Manufacturing & Research, Spain). Until the organ harvest 24 h later, the mice were monitored and scored by a single-blinded investigator. The Evans Blue permeability assay (EB) was conducted as described [11]. In brief, the mice were injected with the 100 µL of 2% wt/vol EB in the tail vein 12 h after LPS injection. For lung cuffing, mice were challenged with LPS (17.5 mg/kg BW) and killed 16 h later and organs were harvested. After the staining process, a lung cuffing score and a percentage of cuffing (yes / no) were raised by a single-blinded person (see Additional file 1: figure S1 and table S2). For Kaplan–Meier survival studies, both models, i.e., LPS and CLP were used. All experiments were performed and analyzed by a blinded investigator.

### Western blot analysis

The Western Blot Analysis was conducted as in [12] described using the SuperSignal™ West Pico Chemiluminescent Substrate (Life Technologies) and Versa Doc Imaging System Model 3000 (BioRad, Hercules, CA) was used to visualize the bands.

### Fluorescent immunocyto-/histochemistry

HUVECs were grown to confluency on Collagen (Sigma-Aldrich, St.Louis, MO) covered coverslips. Antibodies and reagents were used as described above.

Paraffin slices were deparaffined and blocked with 10% donkey serum (Jackson Immuno Research Inc., West Grove, PA). GR-1 was used as primary antibody and pictured with the secondary antibody (goat anti-rat IgG (Alexa flour 555)) (Invitrogen, CA). To evaluate the amount of lung cuffing through scores, mice lungs were stained with Periodic Acid Schiff (PAS) Staining and scored according to Additional file 1: Table S2. The images were taken with the Leica DMI 6000B microscope under the same gain and offset conditions.

### (Micro) RNA isolation and quantitative (q) PCR

For the isolation, the miRNeasy Mini Kit was used (Qiagen, Hilden, Germany) to extract both miRNA and RNA from HUVECs and organ tissue followed by Prime Script RT Reagent Kit (TaKaRa Bio Europe SAS, St Germain-en-Laye, France) after the manufacturer’s instructions.

### Immunoprecipitation of MIR155

HUVECs were plated at 1.8 × 105 cells/well in 6 well-plate overnight and transfected with 200 pM biotinylated control miRNA mimics or 5′-Biotinylated MIR-155 mimics (No. 339178) (Qiagen, Hilden, Germany) using HiPerfect (Qiagen, Hilden, Germany) in accordance with the manufacturer’s instructions. After 48 h of transfection, cells were harvested in 700 µL lysis buffer supplemented with Protease Inhibitor Cocktail (Roche, South San Francisco, CA) and RNaseOUT (Invitrogen) and incubated on ice for 20 min. Lysates were centrifuged at 10,000 × g for 15 min at 4 °C after which 50 µl of the cytoplasmic lysate (input) was transferred into a new tube for RNA extraction. The remaining supernatant was incubated with activated Streptavidin-Dynabeads (Dynabeads M-280 Streptavidin, Invitrogen) for 4 h at 4 °C. After several washing steps and centrifugation, the supernatant was taken for RNA extraction. RNA was subjected to qPCR using Claudin-1 specific primers. The analysis was done as follows: MIRNA pull-down/control pull-down (‘A’), miRNA input/control input (‘B’); fold enrichment = A/B.

### Zebrafish studies

Female Tg(flk1:mCherry) zebrafish were mated with male Tg(l-fabp:eGFP-DPB) to generate Tg(flk1:mCherry/l-fabp:eGFP-DPB) zebrafish offspring. Eggs were microinjected with a MIR155 mimic or a scrambled MIR (mirVana, life technologies) at the one to four cell stage at a concentration of 25 μM [13]. At 96 h post-fertilization, the vascular integrity of the transgenic larvae based on flk1 expression in ECs was determined. In parallel, the plasma protein loss of eGFP-DBP was analyzed by measurement of fluorescence intensity in the retinal vessel plexus [13]. The zebrafish animal studies were performed according to the National Institutes of Health Guideline for the Care and Use of Laboratory Animals. The Mount Desert Island Biological Laboratory (Bar Harbor, ME) animal care committee approved the animal protocol (IACUC protocol #1703). The analysis was performed using ImageJ (Version 1.60, National Institutes of Health, Bethesda, MD) and reported in arbitrary units.

### Transendothelial electrical resistance (TER)

TER was measured using an electric cell-substrate impedance sensing system (ECIS) (Applied BioPhysics Inc.). The continuous values were pooled at discrete time points and plotted versus time. Each conditions’ end point resistance was divided by its starting resistance to give the normalized TER [14].

### Human kidney biopsies

Kidney biopsies were obtained directly post-mortem from patients aged 18 years or older, who died of sepsis. Kidneys from patients diagnosed with kidney cancer who underwent a total nephrectomy, served as controls as described in Aslan et al. Crit. Care. (2014) [15]. The postmortem biopsies were waived by the Medical Ethical Committee of the UMCG, Groningen, The Netherlands (METc 2011/372) [15].

### Expression of MIR155 in human septic serum and bronchoalveolar lavage fluid (BALf) of patients with ARDS

MIR155 expression was measured in serum and BALf samples collected within 24 h of disease onset in patients with ARDS (*n* = 16), with clinical details provided in Table 1 and in healthy controls (*n* = 5). The collection of the BALf and serum samples was approved according to the ethics committee of Hannover Medical School (MHH, EK 8146_BO_K_2018). Total RNA was isolated from human BALf and serum samples using miRNeasy Serum/Plasma Advanced Kit (Qiagen, Cat. No. 217204) following manufacturer’s instructions. For the BALf analysis, we established a novel method base on the above kit used for the serum. Cel_MIR-39 miRNA mimic (Qiagen, Cat. No. 219610) at a concentration of 1.6 × 108 copies/µL was used as a spike-in miRNA control. The reverse transcription for miRNAs was done with equal volume of starting total RNA for each sample and specific TaqMan probes (Applied Biosystems; for MIR-155: Assay ID 002623 and for Cel_MIR-39: Assay ID 000200) using TaqMan MicroRNA Reverse Transcription Kit (Applied Biosystems, Cat. No. 4366597) as per the manufacturer’s guidelines. ViiA7 system (Applied Biosystems) was used to perform miRNAs’ quantification PCR (qPCR) with MIR-155 or Cel_MIR-39 specific TaqMan assays (Applied Biosystems) and Absolute Blue qPCR Mix (Abgene, Cat. No. AB-4136/B). The collection of the BALf and serum samples was approved according to the ethics committee of Hannover Medical School (MHH, EK 8146_BO_K_2018).

**Table 1:** Clinical features of acute respiratory distress syndrome (ARDS) patients

Characteristic	All (*n* = 16)
Age (y), median (IQR)	52 (43–66)
Male (%)	12 (75)
Body mass index (kg/m^2^), median (IQR)	27.7 (24.4–33.3)
Septic shock (%)	9 (56)
Pneumogenic focus (%)	7 (78)
Primary ARDS (%)	14 (88)
*Diagnosis*	
Pneumonia due to	14 (88)
*Influenza A*	4 (29)
*Streptococcus pneumononiae*	4 (29)
*Legionella pneumophilia*	1 (7)
*Staphylococcus aureus* with septicaemia	1 (7)
Unidentified pathogen	4 (29)
Pancreatitis with sepsis and secondary ARDS	1 (6)
Septic shock of unknown focus with secondary ARDS	1 (6)
*Laboratory at time of sampling*	
CRP (mg/L), median (IQR)	252 (134–305)
Procalcitonin (ng/L), median (IQR)	7.7 (1.4–29.1)
Leukocytes (gpt/L), median (IQR)	8.2 (4.8–19.1)
Lactate (mmol/L), median (IQR)	1.6 (1.2–3.2)
p_a_O_2_ / FiO_2_ (mmHg), median (IQR)	109 (77–132)
SOFA score at day of sampling (IQR)	11.5 (8.5–13.0)
*Treatment modalities at time of sampling*	
Invasive ventilation	16 (100)
PEEP (mbar), median (IQR)	14 (12–15)
Pmax (mbar), median (IQR)	26 (23–28)
Extracorporeal membrane oxygenation (%)	7 (44)
Renal replacement therapy (%)	10 (63)
Vasopressor use (%)	13 (81)
Noradrenaline dose (µg/kg/min), median (IQR)	0.085 (0.02–0.35)
28-day ICU-mortality (%)	5 (31)

*ARDS* acute respiratory distress syndrome, *CRP* C-reactive protein, *IQR* interquartile range, *p*_*a*_*O*_*2*_ arterial partial pressure of oxygen, *PEEP* positive end expiratory pressure, *SOFA score* sequential organ failure assessment score. MIR155 expression in relation to cel-MIR39 was lowest in healthy controls (1.03 ± 0.12) versus ARDS patients (12.23 ± 3.0)

### Statistical analysis

Statistical significance was evaluated using Mann–Whitney test or one-way ANOVA unless otherwise noted. All experimental results are presented as mean ± SD, and a two-tailed p value of less than 0.05 was considered to indicate statistical significance. GraphPad Prism 6.0 (La Jolla, CA).

## Results

### Identification of MIRs that are regulated in the endothelium in systemic inflammation

**Fig. 1:**
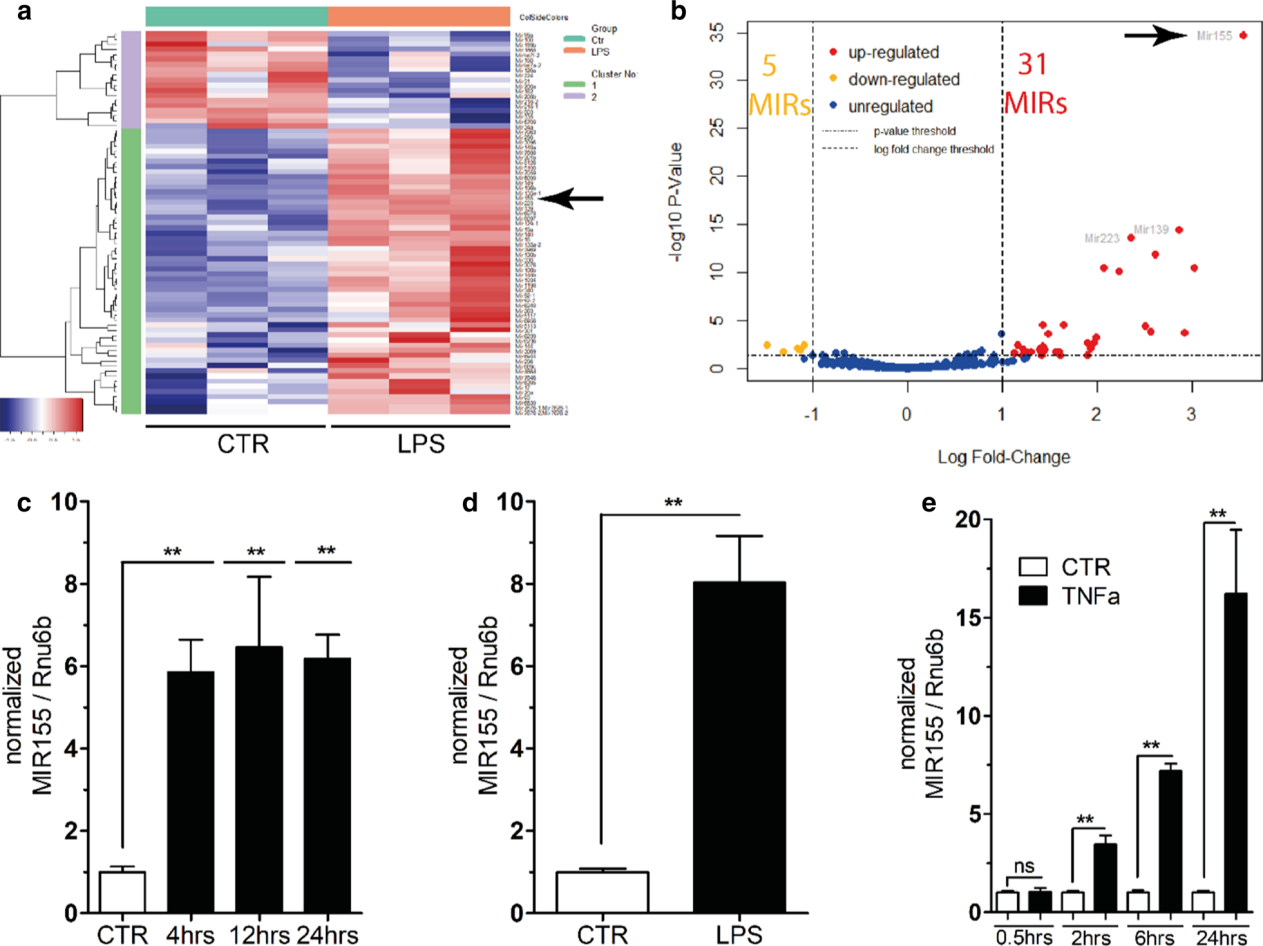
Unbiased MIR screening and confirmation in the endothelium of mice with a systemic inflammatory response. Mice were either given LPS (17.5 mg/kg BW i.p.) or vehicle (0.9% NaCl) and killed after 24 h or at indicated time-points. **a** A smallRNA sequencing was conducted using isolated CD146 + pulmonary endothelial cells (ECs) from endotoxemic compared to healthy mice. Shown is a heat map that highlights the gene distribution with the arrow pointing to the upregulation of MIR155. **b** Volcano plot showing a log scaled distribution of 318 analyzed endothelial MIRs. 5 MIRs were down- and 31 upregulated; MIR155 (arrow) showed the strongest upregulation (**c**). Bar graphs showing normalized MIR155 expression in lung lysates via RT-PCR after 4, 12 and 24 h compared to control (CTR) (CTR: *n* = 8; LPS: *n* = 4–5). **d** Bar graphs showing normalized MIR155/Rnu6b expression in CD146 + pulmonary ECs (*n* = 5). **e** In vitro confirmation of time-dependent MIR155 upregulation in human umbilical endothelial cells (HUVEC) (*n* = 4–6) after stimulation with the proinflammatory cytokine TNFa (50 ng/mL) compared to vehicle (CTR); Bar graphs show mean ± SD of normalized MIR155 with a maximum of 15 × fold increase at 24 h. (**c**–**e**, all **p < 0.01)

Mice were challenged intraperitoneally with a high dosage of LPS (17.5 mg/kg bodyweight, *n* = 3) or vehicle (*n* = 3) to induce a systemic inflammatory response that regularly leads to severe pulmonary capillary leakage. Mice were killed after 24 h, and lungs were harvested for endothelial cell (EC) separation with a magnetic CD146 antibody strategy followed by isolation of MIRs. SmallRNA sequencing was performed together with the Helmholtz Institute (Brunswick, Germany). A heatmap of regulated MIRs of the 6 included animals is shown in Fig. 1a and Additional file 1: Table S1. In summary, out of 318 investigated MIRs, 5 were down- and 31 up-regulated (Fig. 1b and Additional file 1: Table S2). Of note, MIR155—that had not been studied in the septic endothelium so far—showed the strongest signal in our analysis and was therefore followed-up on in the next experiments.

To confirm these earlier findings of the smallRNA seq, MIR155 was first quantified by RT-PCR from whole lung lysates in endotoxemic and control mice at different time-points. Already after 4 h, we found a 6 × fold increase in the LPS-treated mice (Fig. 1c, *p* < 0.01). Given that MIR155 is also expressed by other cells types (e.g., epithelial and immune cells), we selected specifically CD146^+^ pulmonary ECs, which showed an even larger upregulation (i.e., 8 × fold MIR155 increase, Fig. 1d, *p* < 0.01). To confirm these results in a cellular context and to set a system for further mechanistic in vitro studies, confluent HUVEC monolayers were stimulated with 50 ng/mL TNFα (a key cytokine of systemic inflammation) and harvested in a time-dependent fashion. Indeed, TNFα challenge of ECs was sufficient to induce a significant upregulation of the MIR155 already after 2 h (*p* < 0.01) with a maximum response exceeding 15 × fold after 24 h (*p* < 0.001, Fig. 1e).

### Functional consequences of MIR155 overexpression and inhibition in vivo

**Fig. 2:**
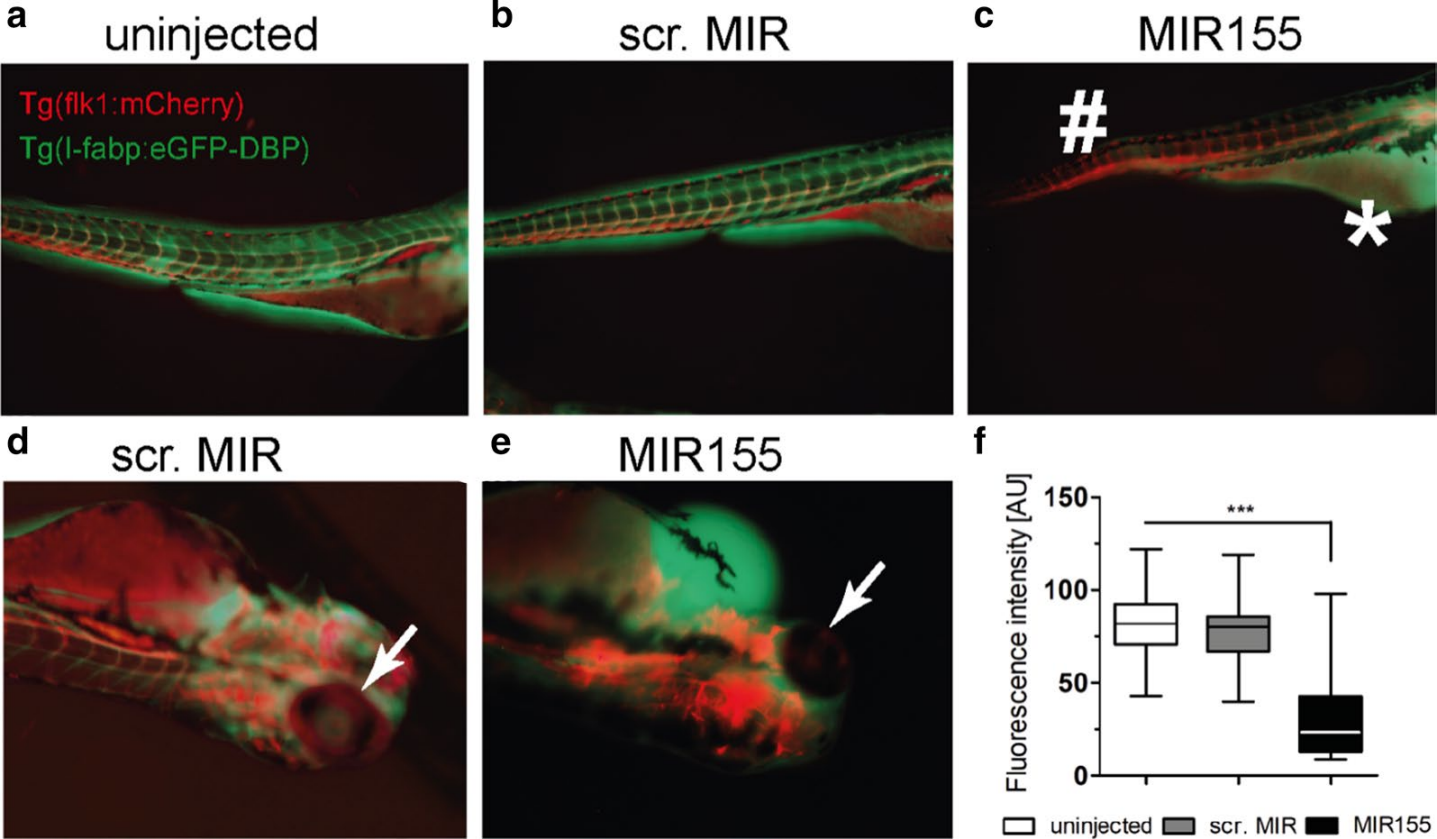
Functional consequences of MIR155 overexpression and inhibition in zebrafish. **a**–**f** Transgenic zebrafish larvae *(Tg(flk1:mCherry)*/*Tg(I-fabp:eGFP-DBP)* were used to assess the role of MIR155 in vivo. Uninjected larvae (*n* = 26), scrambled MIR injected (25 µM, *n* = 16) and MIR155 injected larvae (25 µM, *n* = 26) were analyzed at 96 h post-fertilization. **a** Phenotype analysis shows the eGFP-DBP fusion protein (green) predominantly within the mcherry-flk1 positive vasculature (red) in the uninjected larva. **b** Equivalent eGFP-DBP expression is being detected in the scrambled MIR injection group. **c** MIR155 overexpression leads to loss of eGFP-DBP (green) from the mcherry-flk1 positive vasculature (#) (red) and shows accumulation in the pericardium and yolk sac (*). **d** To quantify protein leakage, the eGFP-DBP content was measured by detection of eGFP-DBP in the retinal vasculature in scrambled and in **e** the MIR155 injected fish. In the retinal vasculature of the MIR155 zebrafish, the fluorescence is diminished as eGFP-DBP is not detectable (arrow). **f** Box and whisker plots showing a quantification of protein leakage that was performed by measurement of the maximum fluorescence intensity of eGFP-DBP in the retinal vasculature indicating a significant loss in the MIR155 injected fish (***p < 0.0001)

Given the lack of knowledge on the functional role of MIR155 in the endothelium, we investigated it in a transgenic Tg(flk1:mCherry)/Tg(l-fabp:eGFP-DBP) zebrafish line. This zebrafish model allowed us (1) to rapidly overexpress MIR155 by microinjection of a MIR155 mimic and (2) to analyze vascular phenotypes. Simplified, this transgenic fish expresses a red cherry protein in the vasculature and has an enhanced green fluorescent protein bound to Vitamin D binding protein (eGFP-DBP) (corresponds to the intravascular compartment, i.e., the plasma). Both loss of GFP intensity in the retinal vessels and the development of pericardial effusion can be used as a surrogate of vascular leak [13, 16]. Indeed, overexpression of MIR155 led to a visually detectable loss of GFP in the red vascular tree and fluid accumulation in the pericardium (Fig. 2a–c). Visualization and quantification of the retinal GFP expression showed a decrease in fluorescence intensity (*p* < 0.0001) supporting the hypothesis that experimental overexpression of MIR155 is sufficient to induce vascular permeability (even in the absence of systemic inflammation) (Fig. 2d–f).

**Fig. 3:**
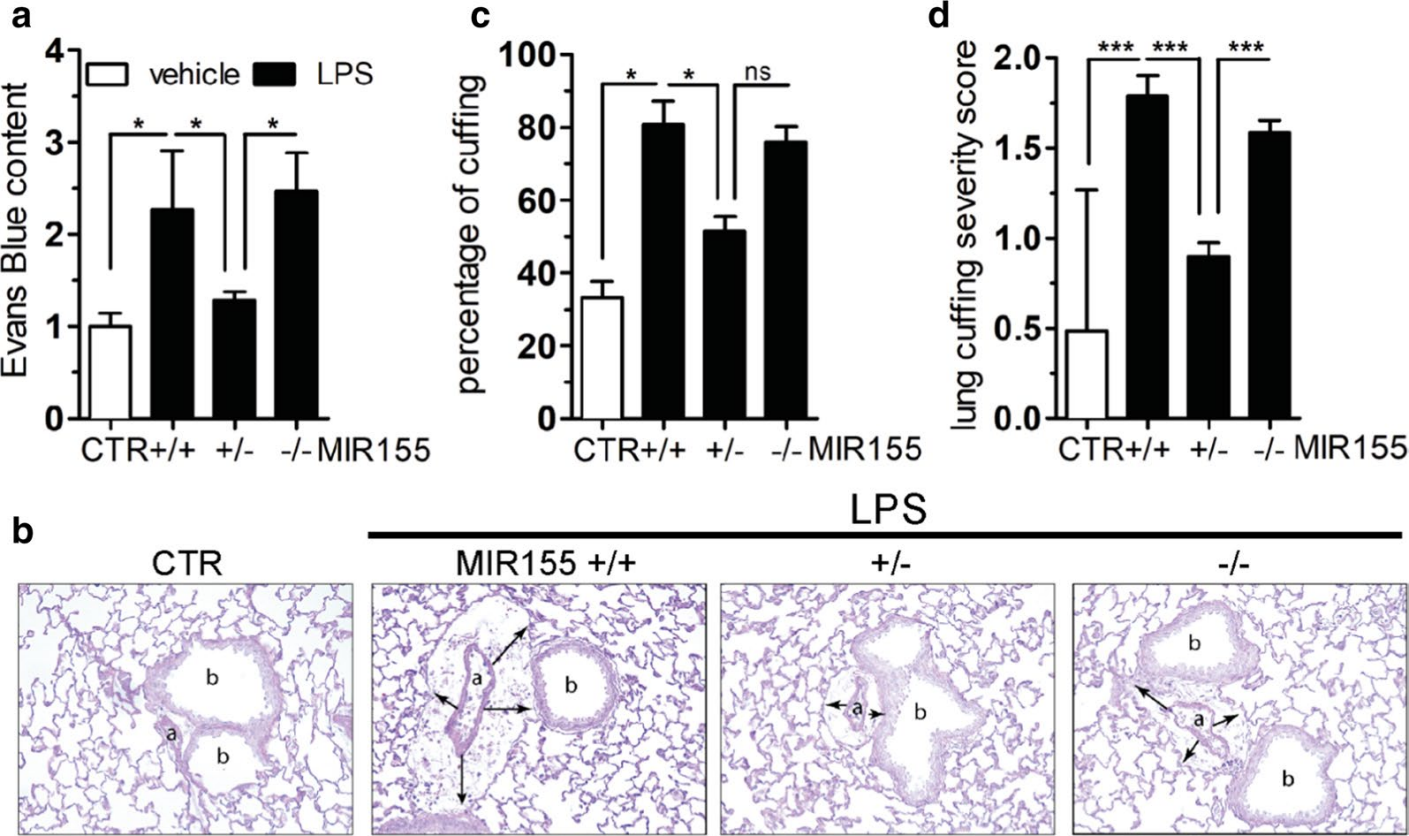
Functional consequences of MIR155 overexpression and inhibition in mice. B6.Cg-Mir155tm1.1Rsky/J knockout mice and wildtype litters were challenged with LPS (17.5 mg/kg BW i.p.) or vehicle (0.9% NaCl) and killed after 16 h. **a** Bar graphs showing Evans blue extravasation in the lung of healthy control mice (CTR, *n* = 5) and LPS challenged B6.Cg-Mir155tm1.1Rsky/J knockout mice (*n* = 3–12) **b**–**d** Perivascular fluid cuffs (lung cuffing) were evaluated. **b** PAS staining indicating the bronchi (b), its corresponding arterial vasa vasorum (a) and cuffing (black arrows). **c** Bar graph showing the percentage of lung cuffing. Indicating a significant difference between the LPS challenged MIR155^+/+^ (*n* = 8) and MIR155^+/−^ group (*n* = 9; *p* = 0.001). **d** Bar graph showing the lung cuffing severity score of single bronchi (*n* = 41–263 arteriolae per group) from a total of 27 mice. (*p < 0.05, **p < 0.01, ***p < 0.0001)

To assess the role of the MIR155 in mammals, knockout mice and littermate wildtypes were challenged with LPS or vehicle and permeability was assessed in vivo by the Evans Blue (EB) method. Septic wildtypes showed a 2.2 × fold increase in lung EB extravasation quantified by spectroscopic analysis of the grounded tissue. Interestingly, homozygote knockouts (MIR155^−/−^) were not protected, but the heterozygote MIR155 mice (MIR155^+/−^) showed barrier properties that were indistinguishable from healthy controls (Fig. 3a). Bronchiolar cuffing is a histological method to visualize pulmonary edema (Additional file 1: Fig. S1). Again, septic wildtype mice showed a severe cuffing phenotype (Fig. 3b, second panel) that was less severe in the heterozygote context (third panel) but not different in the MIR155^−/−^ mice (fourth panel). This finding was semi-quantified by a blinded investigator both with regard to the percentage of cuffing positive bronchi (Fig. 3c) and the severity of cuffing (Fig. 3d). In addition to these observations with regards to permeability, we found additional evidence that the endothelial inflammatory response might be positively influenced if MIR155 is experimentally reduced (Additional file 1: Fig. S2). Together these findings support that MIR155 upregulation in systemic inflammation might be an injurious contributor to vascular leakage across different species.

### Functional consequences of MIR155 overexpression and inhibition in vitro

**Fig. 4:**
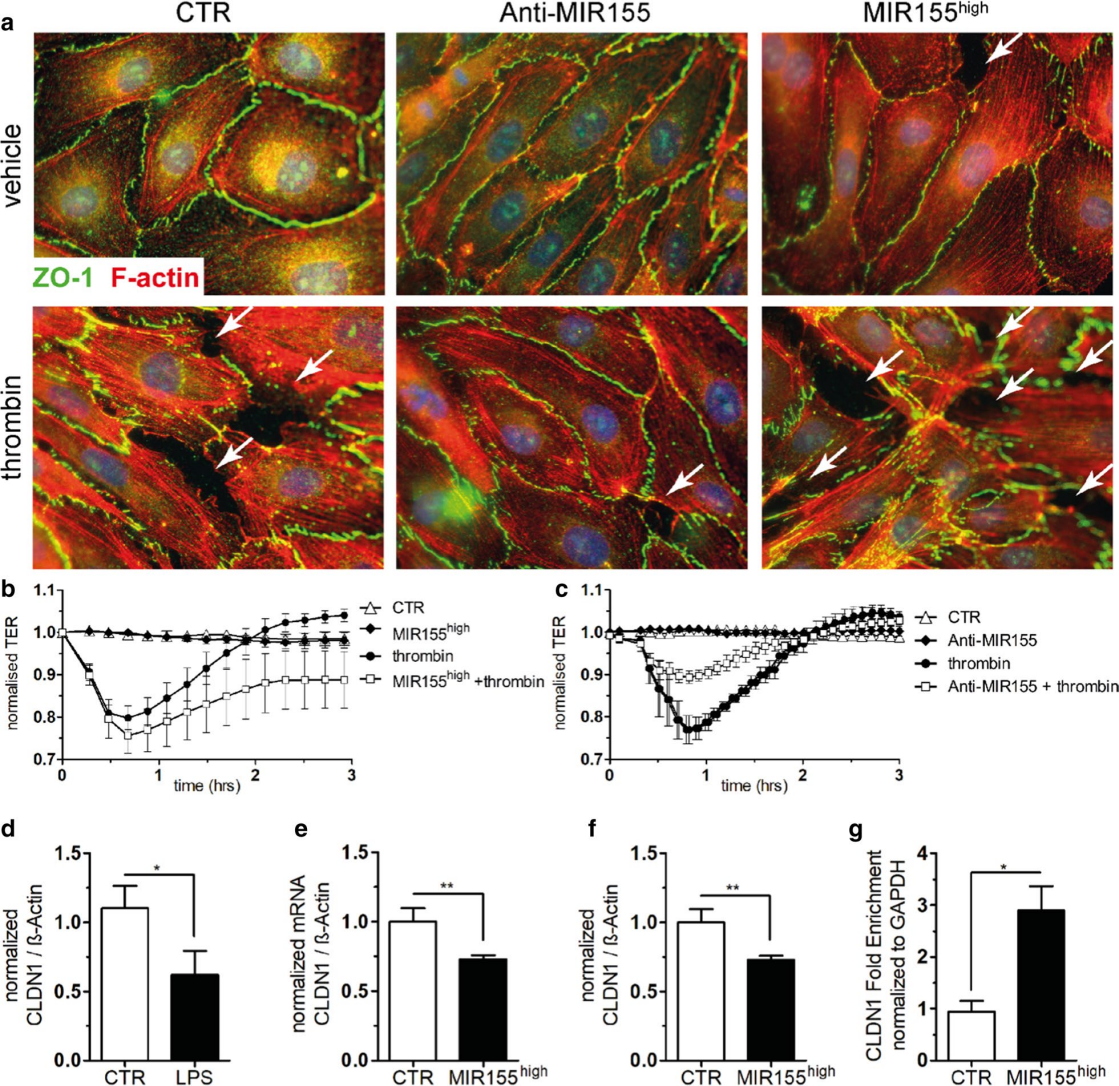
Functional effects of MIR155 on permeability and target candidates. **a** Control, Anti-MIR155 and MIR155^high^ human umbilical vein endothelial cells (HUVECs) were challenged with thrombin or vehicle following staining with a tight-junction protein (ZO-1, green), the cytoskeleton (F-actin, red) and DAPI (blue). Thrombin challenged ECs showed visible gap formation between adjacent cells (white arrows). Blockade of MIR155 had visually lesser gap formations upon thrombin stimulation (lower middle panel). Whereas the MIR155^high^ transfected HUVECs showed mild spontaneous (upper right) and severe gap formation after thrombin stimulation (lower right). **b** MIR155 transfected HUVECS (MIR155^high^) were grown under constant detection of the transendothelial resistance (TER) until confluency was reached. Challenge with thrombin showed a stronger decrease and slower recovery (endpoint *p* < 0.05) in MIR155^high^ compared to naïve ECs. **c** Continuous TER between vehicle and Anti-MIR155 challenged with thrombin showed an amelioration of maximal response and faster re-bound recovery than the corresponding control group (deepest drop point *p* < 0.05) **d** Densitometry from C57BL/6J mouse lungs challenged with either LPS (17.5 mg/kg BW, *n* = 5) or vehicle (NaCl 0.9%, *n* = 7). After 16 h they were killed and Claudin-1 (CLDN-1) and b-Actin were detected by immunoblotting (*p* < 0.05). **e** Bar graphs showing normalized mRNA of Claudin-1/bActin in MIR155^high^ and naïve (CTR) HUVECs (*n* = 6–7; *p* < 0.01). **f** Bar graphs showing densitometry results of Claudin-1/bActin immunoblots in MIR155^high^ versus naïve (CTR) HUVECs (*n* = 6–7; *p* < 0.01). **g** Bar graphs showing immunoprecipitation of the Claudin-onefold enrichment normalized to GAPDH (*n* = 4, *p* < 0.05), after the overexpression of MIR155 in HUVECs (*n* = 4, *p* < 0.05)

In vitro, naïve or MIR155 modulated HUVECs were challenged with thrombin (a mediator of systemic inflammation and an established inductor of EC permeability in vitro) to recapitulate the above described in vivo findings. Thrombin stimulation primed the formation of F-actin polymerization and gap formation between adjacent ECs leading to an increase in permeability (white arrows) (Fig. 4a, lower left). ECs that have been challenged with a MIR mimetic (w/o thrombin) partly phenocopied these permeability patterns (Fig. 4a, upper right). Interestingly, from a therapeutic point of view, ECs that have been co-stimulated with thrombin + anti-MIR155 peptide were protected from the development of the before described endothelial morphological changes (Fig. 4a, lower middle panel). To reliably quantify these qualitative changes in morphology, we measured TER in real-time with Electric Cell-substrate Impedance Sensing (ECIS). MIR155 overexpression was not sufficient to induce leak in this assay by itself, but MIR155 overexpression critically interfered with the usual recovery after stimulation with thrombin (Fig. 4b). More important from a translational aspect, inhibition of MIR155 (anti-MIR155) was sufficient to reduce the deleterious effect of thrombin in this highly sensitive assay (Fig. 4c, *p* < 0.05). Together these findings support our hypothesis that upregulation of MIR155 in the septic endothelium is indeed injurious and that its inhibition might represent a therapeutic target.

### Identification of MIR155 target candidates

Using an in silico analysis tool (http://www.targetscan.org), we identified 556 potential transcripts with preserved sites among various species that might be influenced by MIR155. Given the permeability phenotype in our in vivo and in vitro models, we specifically looked at proteins involved in the junctional apparatus. The analysis predicted that Claudin-1 could be a possible target that additionally showed conserved binding sites among different species (e.g., human, cow, rat, mouse, dog). First, we checked if Claudin-1 expression is indeed altered in LPS-treated mice and found a significant reduction in their lungs (Fig. 4d). Experimental up-regulation of MIR155 in ECs in vitro to a similar extent that we earlier detected in systemic inflamed mice (Additional file 1: Fig. S3) induced a significant downregulation of Claudin-1 on mRNA and protein levels (Fig. 4e, f). Using an immunoprecipitation strategy from ECs stimulated with an MIR155 mimetic, we could confirm the physical binding of MIR155 with claudin-1 (Fig. 4g). However, inhibition of MIR155 was not sufficient to increase Claudin-1 expression spontaneously (data not shown).

### Effect of MIR155 depletion on clinical outcomes in murine sepsis

**Fig. 5:**
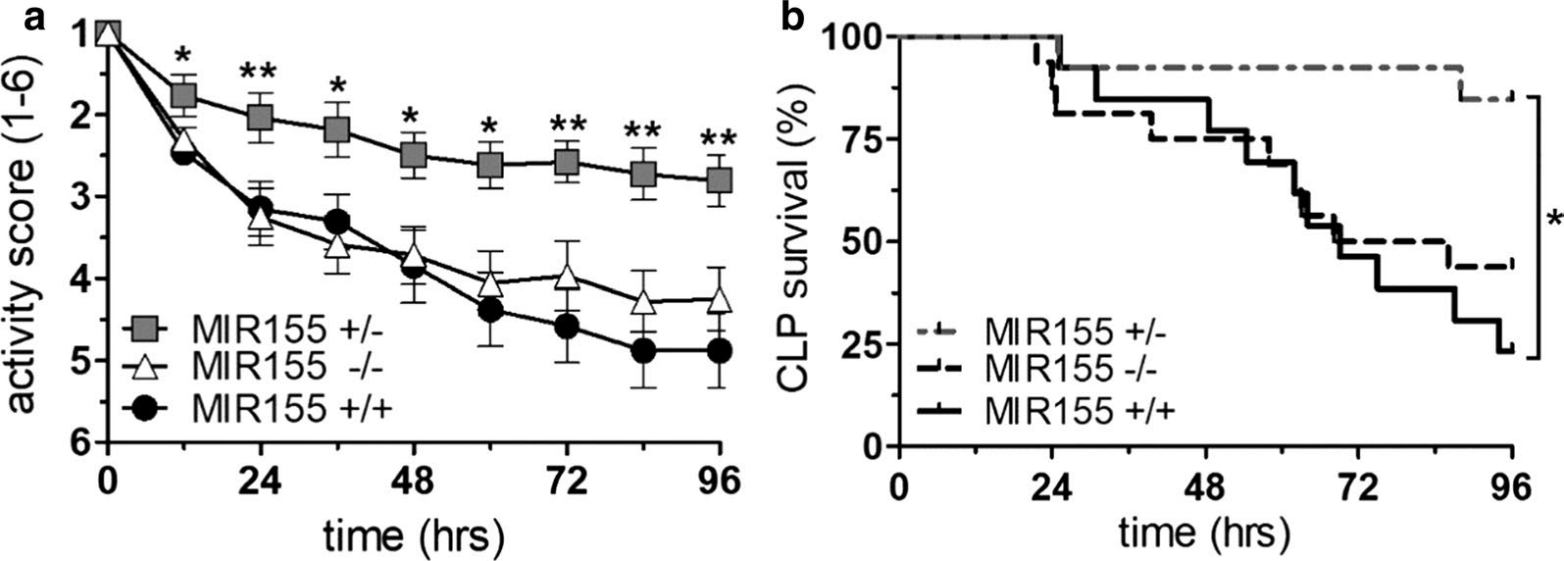
Effect of MIR155 depletion on clinical outcomes in murine sepsis. **a** Box and whiskers showing a descending activity score (explained in Additional file 1: Table S2.) that is attenuated in the heterozygote background with a significant difference at all time points calculated by Kruskal–Wallis test (*p < 0.05, **p < 0.001). **b** Kaplan–Meier-survival analysis of mice suspected to cecal ligature & puncture (CLP) showing improved outcome in heterozygous knockout mice (*p* = 0.0135 with Mantel–Cox test)

Next, we tested if the previously observed attenuation of permeability in the MIR155 heterozygote mice might be sufficient to protect from organ dysfunction and death. Analysis of functional organ parameters from serum samples 24 h after LPS administration in all genotypes showed trends towards improved organ function and survival in a 100% lethal model (Additional file 1: Table S3 and Fig. S3.) in the heterozygous MIR155^+/−^ mice (median survival MIR155^+/−^ 25.5 h vs MIR155^+/+^ 36 h). The 100% lethality of our endotoxemia model combined with increasing doubts regarding the clinical relevance of this model in the sepsis community [17] inspired us to test outcome in a clinically more meaningful polymicrobial sepsis model, i.e., CLP. Upon CLP surgery, mice were regularly scored for severity of disease by a single-blinded investigator using a standardized activity score (Additional file 1: Table S4). Consistent with the permeability data, MIR155^+/−^ mice had a better physical performance indicating a less severe disease manifestation than all other groups over a 96 h observation period (Fig. 5a) and indeed showed a clear survival benefit by approximately 60% compared to the MIR155^+/+^ mice. (Mantel–Cox Test, *p* < 0.05, Fig. 5b).

### Translational evidence of MIR155 regulation in human disease

**Fig. 6:**
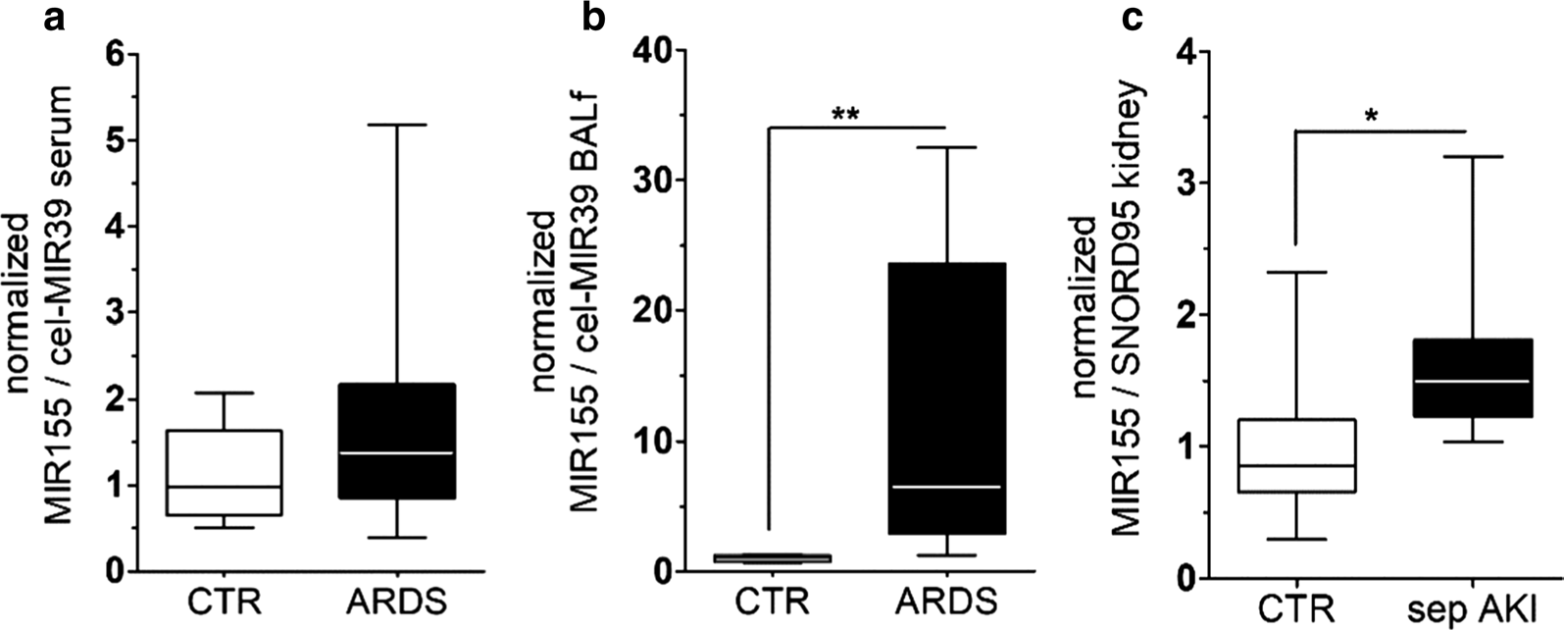
Translational evidence of MIR155 regulation in human disease. **a** Box and whisker plots showing normalized circulating MIR155 levels in serum of healthy controls (CTR, *n* = 5) and patients with acute respiratory distress syndrome (ARDS, *n* = 16) **b** Box and whisker plots showing normalized MIR155 in bronchoalveolar lavage fluid (BALf) of the same patients as in **a** (**p < 0.001), **c** Box and whisker plots showing normalized MIR155 in immediate postmortem kidney biopsies from healthy control biopsies (CTR, *n* = 8) and septic acute kidney injuries (sep AKI, *n* = 12) (*p = 0.02)

Given the discovery of both cross-species regulation and relevance of MIR155 in increased endothelial permeability, we set out to investigate its regulation in a human organism. To do this, we followed two strategies. First, we analyzed BALf and serum from critically ill patients suffering from ARDS—a syndrome that is pathophysiologically based on increased vascular permeability. Patients characteristics are summarized in Table 1. To our surprise, circulating MIR155 levels were not different between controls and ARDS patients **(**Fig. 6a). Given that we identified MIR155 with the RNA seq specifically in the pulmonary endothelium, we analyzed local MIR155 abundance in the alveoli (i.e., BALf) of healthy controls and ARDS patients. The healthy volunteers (*n* = 5) had a median age of 26.9 years [IQR 22.5–33.1] and were all male. Consistent with our previous findings, we found a massive MIR155 upregulation in ARDS patients in this local compartment, close to the pulmonary endothelium (Fig. 6b). To test MIR155 regulation in other organs frequently affected during sepsis, we analyzed immediate post-mortem kidney biopsies from patients with septic acute kidney injury compared to healthy parts of tumor nephrectomy samples. Again, we could confirm that MIR155 was significantly upregulated in human septic kidneys (Fig. 6c). Together, these findings highlight the translational relevance of our results.

## Discussion

Using an unbiased small RNA sequencing approach, we found that MIR155 was highly upregulated in the pulmonary endothelium of endotoxemic mice. MIR155 overexpression was sufficient to induce spontaneous vascular leakage in zebrafish, augment pathological responses in cultured human endothelial cell monolayers and exacerbate the endothelial inflammatory response in murine sepsis. Moreover, pharmacological inhibition or genetic depletion of MIR155 prevented breakdown of the vascular barrier and improved global outcome in animal models of systemic inflammation and sepsis. Finally, MIR155 was also detectable in diverse human samples highlighting the potential translational relevance of our experimental findings.

In general, the role of MIRs in endothelial response to systemic inflammation is not well understood. To our knowledge, only a single study has linked a panel of MIRs (including MIR155) to endothelial inflammation [18], whereas most MIR155 research has focused on T-cell biology [19, 20]. Three independent reports support our investigation of MIR155 in the inflamed vasculature. First, Han et al. reported increased circulating MIR155 levels in human sepsis [21]. Second, Pena-Philippides et al. implicated MIR155 in the regulation of endothelial tight junctions after cerebral ischemia [22]. Specifically, they reported that inhibition of MIR155 fortified monolayers of human primary brain microvascular endothelial cells against barrier breakdown following oxygen–glucose deprivation. Studying lung microvascular ECs, Pfeiffer, et al., concluded that inhibition of MIR155 could blunt microvascular endothelial expression of inflammatory mediators [18].

Given that our transcriptomics approach in endotoxemic mice revealed MIR155 upregulation, we first sought to clarify if elevated endothelial MIR155 was injurious or adaptive. Transparency of larval zebrafish and ease of gene transduction makes this model amenable to studying vascular phenotypes. Overexpression of MIR155 indeed revealed compromised vascular integrity in two different assays, supporting the conclusion that excess MIR155 may be alone be injurious and may potentiate adverse responses to the septic milieu. Next, we investigated a B6.Cg-Mir155tm1.1Rsky/J knockout mouse to (1) test whether MIR155 was required for a severe inflammatory response; (2) evaluate the MIR155 hypothesis in a mammalian species; and (3) explore its relevance in models of human disease. Although endotoxemia is not a model of sepsis per se*,* the consequences of sterile cytokine storm are still of translational interest, and this noxious stimulus is an established trigger of pulmonary vascular hyperpermeability, a key phenotype of interest [23, 24]. LPS strain and dose were tailored in a pilot study to induce significant pulmonary edema early in cytokine storm rather than to evaluate overall survival. To study survival, we applied the murine gold standard sepsis model of CLP (that was piloted to a mortality of 75%) to MIR155 knockouts.

The absence of a gene-dose effect—namely that heterozygous mice were protected, whereas null mice were not—was unexpected. However, our group has observed similar results in the context of Angiopoietin-2, another regulator of endothelial permeability [25]. Several hypotheses are possible. First, there might be a subtle developmental effect of complete gene loss that is exacerbated by the stress of sepsis—in the case of Angiopoietin-2, null mice have marked lymphedema from aberrant lymphangiogenesis. Second, compensatory developmental and/or physiological mechanisms may become activated with complete gene loss. For example, compensatory upregulation of MIR146a could be implicated in MIR155 knockouts [18]. However, we did not find such an association with MIR155 gene dose (data not shown). Therefore, future experiments will be required in order to explore the lack of protection against sepsis exhibited by MIR155 knockout mice.

Different compartments (lung, kidney, serum) were analyzed in the human components of this study in order to parse local and systemic regulation of MIR155. First, we compared bronchoalveolar lavage (BAL) fluid and serum samples from patients with ARDS versus healthy controls. Although reported by others [26], we did not observe a significant change in circulating MIR155 among ARDS patients. Yet, these very same patients showed clear MIR155 elevation in BAL samples. While this difference may be attributable to underlying patient and disease characteristics, the presence of a BAL-specific difference in MIR155 concentration raises the possibility of organ-specific actions of this microRNA. The human kidney data also suggest regional regulation of MIR155. However, results from these post-mortem specimens may be difficult to interpret as they represent the end stage of sepsis with multiple organ failure. On the other hand, biopsies from failing organs in sepsis are extremely challenging to acquire, and the biopsy itself could be harmful with limited potential benefit to the individual patient.

Our study has important limitations. The MIR155 knockout model was neither conditional nor organ-specific. Therefore, improvement in overall survival following CLP may be partially attributable to non-endothelial effects. The observation that heterozygosity but not complete gene depletion protects from endothelial injury requires further investigation. Finally, the underlying molecular mechanisms regulating MIR155 merit future study.

## Conclusions

We found cross-species evidence in knockout mice, transgenic zebrafish, human endothelial cells and critically ill patients that MIR155 is upregulated in the endothelium during systemic inflammation. MIR155 upregulation may lead to vascular barrier breakdown by targeting the tight-junction protein Claudin-1. Conversely inhibition of MIR155 may improve vascular integrity and overall survival, making this microRNA a putative therapeutic target for further exploration in sepsis and related indications. Future experimental studies are needed to understand the regulation of MIR155 and to elucidate its downstream effects in multiple cell types.

## Supplementary Information

**Additional file 1:** Supplemental Material.

## Data Availability

All data are stored and available upon reasonable request at the laboratory of Professor David at the Hannover Medical School, Germany.
