# Supplementary material for: Role of endothelial microRNA 155 on capillary leakage in systemic inflammation
Source: Crit Care. 2021 Feb 22;25:76. doi: 10.1186/s13054-021-03500-0 (PMC7901081; doi:10.1186/s13054-021-03500-0)
Supplement: Supplementary file 1 — Additional file 1: Supplemental Material. [file 13054_2021_3500_MOESM1_ESM.docx]

**SUPPLEMENTAL FIGURES**

**Supplemental Figure 1.** Lung cuffing score to evaluate the severity.


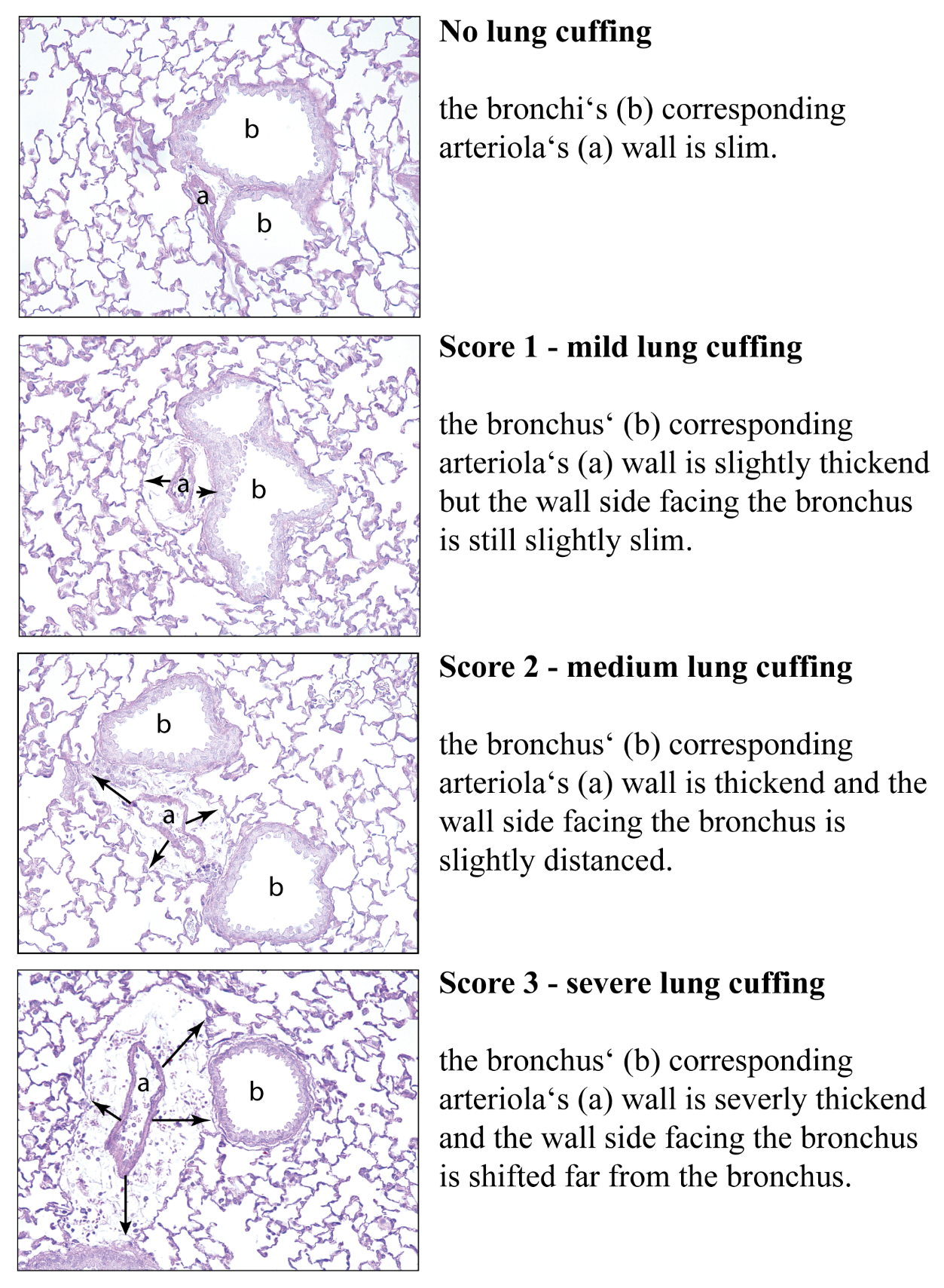


**Supplemental Figure 2.** Endothelial inflammatory response might be reduced in heterozygous MIR155 mice.


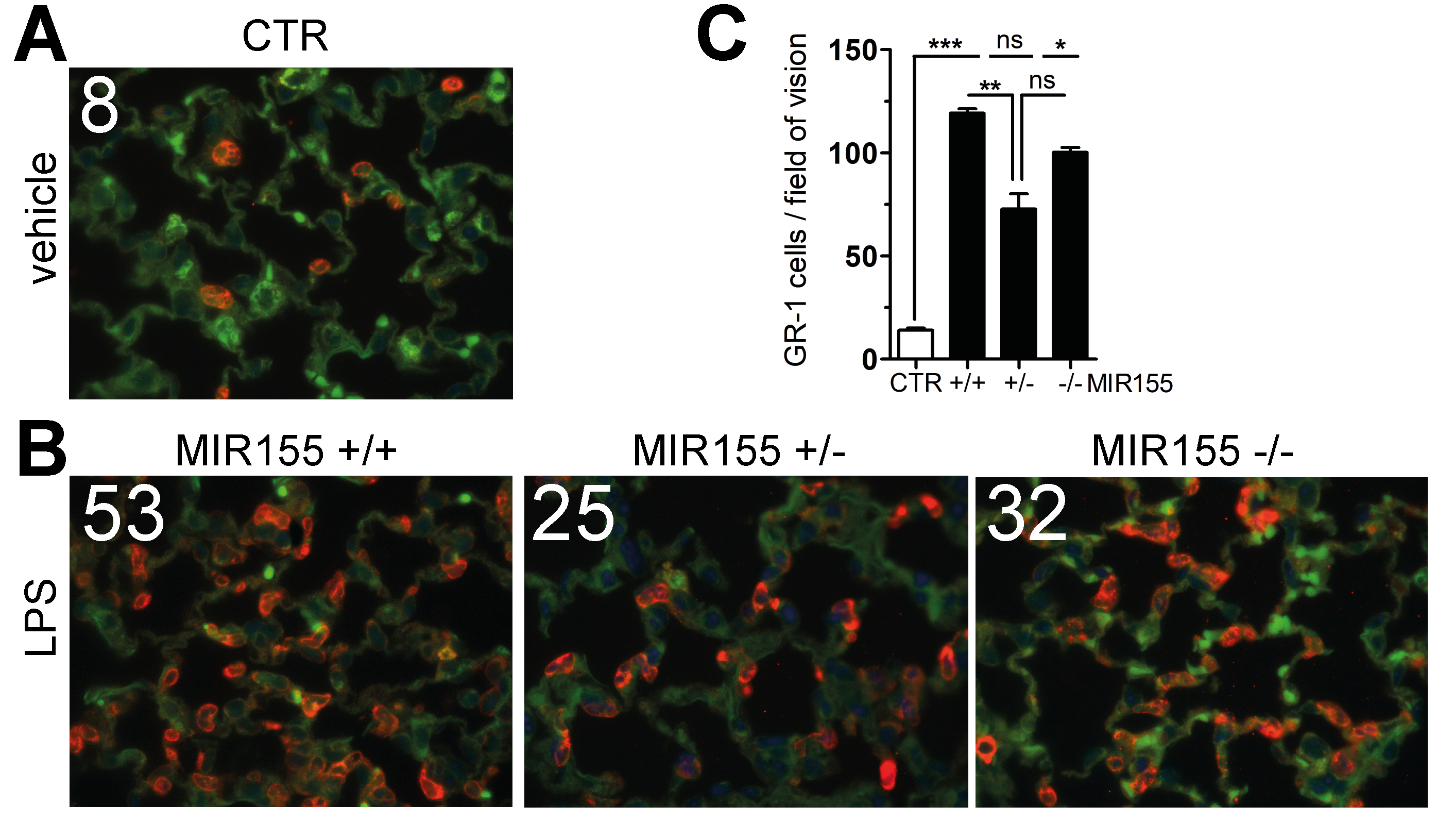


B6.Cg-Mir155tm1.1Rsky/J knockout mice were challenged with LPS (17.5mg/kg BW i.p.) or vehicle (0.9% NaCl) and sacrificed after 16 hours. **(A, B)** Mice lungs were stained with immunofluorescent GR-1 (red) and DAPI (blue). Pictures were taken with a self-fluorescent background highlighting the lung tissue (green). **(A)** Immunofluorescent picture showing the control group (vehicle) with 8 GR-1 positive cells (white number). **(B)** Immunofluorescent pictures were taken of LPS challenged MIR155 +/+, +/- and -/- mice with a counted amount of GR-1 positive cells (white number). **(C)** Bar graph showing the number of GR-1 positive cells in mice lungs per field of vision. Indicating that the heterozygous MIR155^+/-^ mice (n=8, per 10 fields of vision per mouse) were having a less stronger inflammatory reaction compared to homozygous MIR155^+/+^ mice (n=5, per 10 fields of vision per mouse, p<0.05) or the homozygous MIR155 ^-/-^ mice (n=8, per 10 fields of vision per mouse, p>0.05).

**Supplemental Figure 3. Overexpression of MIR155 in endothelial cells.**
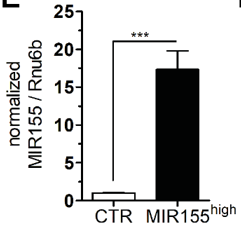


Bar graphs showing the extent of MIR155 overexpression in MIR155^high^ HUVECS (n=8) by the approximately similar fold change compared to TNFa stimulation (***p<0.001).

**Supplemental Figure 4.** Survival in endotoxemic mice dependent on specific MIR155 genotype.





Kaplan-Meier-curve showing murine LPS survival in B6.Cg-Mir155tm1.1Rsky/J mice which were regularly checked. The median survival in the MIR155^+/-^ was 10 hrs longer compared to MIR155^+/+^ and MIR155^-/-^ mice.

**Supplemental Table 1.** Regulation of MIRs

A smallRNA sequencing was conducted using isolated CD146^+^ pulmonary endothelial cells (ECs) from endotoxemic compared to healthy mice. The table shows the fold regulation of individual testes of all the genes whose p-values are smaller than 0.05 (n=75).

| Name of MIR | P-value | logFC | FDR |
| --- | --- | --- | --- |
| Mir155 | 2,1562E-08 | 3,54570979 | 6,8566E-06 |
| Mir139 | 3,6142E-06 | 2,87161889 | 0,00046504 |
| Mir223 | 4,5429E-06 | 2,36024227 | 0,00046504 |
| Mir135a-1 | 5,8496E-06 | 2,61416664 | 0,00046504 |
| Mir149 | 9,5591E-06 | 2,07698095 | 0,00060796 |
| Mir340 | 1,5179E-05 | 2,23313741 | 0,00080447 |
| Mir135a-2 | 2,0249E-05 | 3,02903488 | 0,00091989 |
| Mir6978 | 0,00023405 | 2,92567294 | 0,00792713 |
| Mir196b | 0,00024343 | 2,50531058 | 0,00792713 |
| Mir18 | 0,00024928 | 1,42679093 | 0,00792713 |
| Mir5099 | 0,00029215 | 0,99610743 | 0,00844567 |
| Mir129-1 | 0,00034132 | 1,64391452 | 0,00904498 |
| Mir6240 | 0,00064178 | 1,49366675 | 0,01569881 |
| Mir301b | 0,00080158 | 2,57154868 | 0,01812075 |
| Mir8097 | 0,00085475 | 1,99899463 | 0,01812075 |
| Mir5100 | 0,00207094 | 1,17030054 | 0,04040671 |
| Mir218-1 | 0,00216011 | -1,08733867 | 0,04040671 |
| Mir1198 | 0,00291252 | 0,78544119 | 0,0498947 |
| Mir6236 | 0,00306643 | 1,89791134 | 0,0498947 |
| Mir6539 | 0,0033452 | 1,96611136 | 0,0498947 |
| Mir298 | 0,00335189 | 1,43284326 | 0,0498947 |
| Mir126a | 0,00357199 | -1,47906643 | 0,0498947 |
| Mir182 | 0,00423067 | -1,15831097 | 0,0498947 |
| Mir218-2 | 0,00425658 | -1,11732291 | 0,0498947 |
| Mir363 | 0,00437029 | 1,39041041 | 0,0498947 |
| Mir100 | 0,00437032 | -0,66223767 | 0,0498947 |
| Mir106b | 0,00447385 | 0,75891576 | 0,0498947 |
| Mir503 | 0,00458124 | -0,7508536 | 0,0498947 |
| Mir1934 | 0,00465467 | 1,25877874 | 0,0498947 |
| Mir130b | 0,00470705 | 1,22494171 | 0,0498947 |
| Mir7059 | 0,00558716 | 1,45223607 | 0,05488987 |
| Mir148b | 0,00568023 | 0,71048456 | 0,05488987 |
| Mir5126 | 0,00569612 | 1,94011435 | 0,05488987 |
| Mir92-2 | 0,0062312 | 0,64241439 | 0,05828001 |
| Mir3096 | 0,0075345 | 0,64420969 | 0,06845628 |
| Mir3076 | 0,00798973 | 1,3007843 | 0,07057599 |
| Mir140 | 0,0082209 | 0,54979528 | 0,07065528 |
| Mir5709 | 0,00927719 | -1,30856728 | 0,0776299 |
| Mir92-1 | 0,00971314 | 0,61414139 | 0,0776299 |
| Mir99a | 0,00976477 | -0,56709137 | 0,0776299 |
| Mir3969 | 0,01023302 | 1,12992937 | 0,07936831 |
| Mir200a | 0,01136243 | -0,89594961 | 0,08602982 |
| Mir19a | 0,01282712 | 0,91672664 | 0,09486106 |
| Mir31 | 0,01332801 | -0,99779978 | 0,09632513 |
| Mir7676-2 | 0,01440156 | 1,60055709 | 0,10177102 |
| Mir7676-1 | 0,01523931 | 1,56783293 | 0,10535005 |
| Mir335 | 0,01560899 | -0,86817928 | 0,10560979 |
| Mir93 | 0,01634548 | 0,54737976 | 0,10725645 |
| Mir6956 | 0,01691084 | 1,41474967 | 0,10725645 |
| Mir669c | 0,01705789 | 1,20983948 | 0,10725645 |
| Mir150 | 0,01720151 | -0,65793392 | 0,10725645 |
| Mir148a | 0,01876147 | 0,52286711 | 0,11473363 |
| Mir7646 | 0,02134227 | 1,61303418 | 0,12805361 |
| Mir144 | 0,02280147 | 1,89401393 | 0,13427533 |
| Mir6944 | 0,02397137 | 0,91134385 | 0,13859811 |
| Mir296 | 0,02562241 | 0,92875126 | 0,14313683 |
| Mir6239 | 0,0256566 | 0,89110854 | 0,14313683 |
| Mir330 | 0,02689791 | 0,70617529 | 0,14747472 |
| Mir301 | 0,02839817 | 0,69183737 | 0,1530613 |
| Mir466d | 0,0306835 | 1,2338045 | 0,16262257 |
| Mir5113 | 0,03241015 | 1,25951028 | 0,16484555 |
| Mir20a | 0,03246045 | 0,6359557 | 0,16484555 |
| Mir17 | 0,03265808 | 0,56439049 | 0,16484555 |
| Mir6395 | 0,03418885 | 1,12098849 | 0,16792774 |
| Mir208b | 0,03432485 | -1,08385007 | 0,16792774 |
| Mirlet7f-2 | 0,03548092 | -0,54877321 | 0,17023594 |
| Mir224 | 0,03613975 | -0,84229815 | 0,17023594 |
| Mirlet7a-2 | 0,03686922 | -0,47925176 | 0,17023594 |
| Mir7063 | 0,03693799 | 1,00303449 | 0,17023594 |
| Mir1668 | 0,03811529 | -0,69139855 | 0,17315234 |
| Mir34a | 0,04092798 | -0,60997578 | 0,18223807 |
| Mir199b | 0,04126145 | -0,54086484 | 0,18223807 |
| Mir3069 | 0,04462449 | 1,06238282 | 0,19439159 |
| Mir5117 | 0,04609383 | 0,54288302 | 0,19694942 |
| Mir7688 | 0,04645034 | 0,59887622 | 0,19694942 |

**Supplemental Table 2** Regulation of MIRs

A smallRNA sequencing was conducted using isolated CD146^+^ pulmonary endothelial cells (ECs) from endotoxemic compared to healthy mice. The table shows the fold regulation of individual testes of all the genes whose FDR is smaller than 0.05 and it logFC is <-1.

| Name of MIR | P-value | logFC | FDR |
| --- | --- | --- | --- |
| Mir155 | 6,0765E-38 | 3,54568589 | 1,9323E-35 |
| Mir139 | 2,2768E-17 | 2,87162658 | 3,6201E-15 |
| Mir223 | 2,5394E-16 | 2,3602423 | 2,6918E-14 |
| Mir135a-1 | 1,7344E-14 | 2,61506479 | 1,3788E-12 |
| Mir135a-2 | 6,4151E-13 | 3,02982609 | 3,9664E-11 |
| Mir149 | 7,4838E-13 | 2,07698802 | 3,9664E-11 |
| Mir340 | 1,9517E-12 | 2,23312952 | 8,8664E-11 |
| Mir18 | 8,3033E-07 | 1,42719931 | 3,1668E-05 |
| Mir129-1 | 8,9626E-07 | 1,64408994 | 3,1668E-05 |
| Mir196b | 1,3557E-06 | 2,50608192 | 4,311E-05 |
| Mir301b | 5,0383E-06 | 2,57176003 | 0,00014565 |
| Mir6978 | 7,3061E-06 | 2,9243652 | 0,00019361 |
| Mir6240 | 9,6797E-06 | 1,49368833 | 0,00023678 |
| Mir5099 | 1,0525E-05 | 0,99611233 | 0,00023906 |
| Mir8097 | 2,8113E-05 | 1,99878462 | 0,000596 |
| Mir6539 | 0,00012981 | 1,96584822 | 0,00257996 |
| Mir6236 | 0,00014048 | 1,89811943 | 0,00262771 |
| Mir5100 | 0,00023244 | 1,17030334 | 0,00410647 |
| Mir126a | 0,00025287 | -1,47771258 | 0,00423232 |
| Mir218-1 | 0,00028119 | -1,08730802 | 0,00447085 |
| Mir298 | 0,000359 | 1,4324097 | 0,0054363 |
| Mir182 | 0,00064205 | -1,15830898 | 0,00928049 |
| Mir5126 | 0,00069289 | 1,93720123 | 0,00957996 |
| Mir218-2 | 0,0008008 | -1,11733135 | 0,0104268 |
| Mir130b | 0,00081972 | 1,22485822 | 0,0104268 |
| Mir363 | 0,00087187 | 1,39035773 | 0,01066358 |
| Mir7059 | 0,00091097 | 1,45200486 | 0,01072919 |
| Mir1198 | 0,00144883 | 0,78554534 | 0,01645452 |
| Mir1934 | 0,00171202 | 1,26069082 | 0,01877318 |
| Mir5709 | 0,00195551 | -1,30845396 | 0,02072841 |
| Mir7676-2,Mir7676-2 | 0,00207283 | 1,60007581 | 0,02126323 |
| Mir7676-1,Mir7676-1 | 0,00231742 | 1,56762779 | 0,02226545 |
| Mir106b | 0,00234753 | 0,75893382 | 0,02226545 |
| Mir3076 | 0,00238058 | 1,30026294 | 0,02226545 |
| Mir503 | 0,00255622 | -0,75093656 | 0,02322511 |
| Mir100 | 0,00295833 | -0,66224836 | 0,0261319 |
| Mir3969 | 0,00339239 | 1,12982901 | 0,0291562 |
| Mir148b | 0,0035776 | 0,71049357 | 0,02993884 |
| Mir92-2 | 0,00470384 | 0,64241244 | 0,03835442 |
| Mir19a | 0,00533051 | 0,91726604 | 0,04237753 |
| Mir200a | 0,00558245 | -0,89598202 | 0,04294802 |
| Mir31 | 0,00580602 | -0,9979958 | 0,04294802 |
| Mir92-1 | 0,00580744 | 0,6141412 | 0,04294802 |
| Mir669c | 0,0063512 | 1,20883346 | 0,04493826 |
| Mir144 | 0,00635919 | 1,89805285 | 0,04493826 |
| Mir3096 | 0,0069095 | 0,64424948 | 0,04730808 |
| Mir6956 | 0,00699207 | 1,41493244 | 0,04730808 |
| Mir7646 | 0,00721346 | 1,61060112 | 0,04778915 |
| Mir335 | 0,00837598 | -0,86811934 | 0,05435843 |
| Mir140 | 0,00875503 | 0,54979625 | 0,05568198 |
| Mir99a | 0,01113044 | -0,56710246 | 0,06940158 |
| Mir150 | 0,01301245 | -0,65793505 | 0,07957612 |
| Mir5113 | 0,01422782 | 1,25811495 | 0,08536695 |
| Mir6239 | 0,01499514 | 0,89109844 | 0,08732011 |
| Mir466d | 0,01510254 | 1,23358368 | 0,08732011 |
| Mir296 | 0,01599669 | 0,92874008 | 0,09000619 |
| Mir6944 | 0,01613319 | 0,9095845 | 0,09000619 |
| Mir93 | 0,01743267 | 0,54738279 | 0,09557913 |
| Mir208b | 0,01912654 | -1,08557651 | 0,10308879 |
| Mir330 | 0,02152579 | 0,70622381 | 0,11347891 |
| Mir148a | 0,02176797 | 0,52286811 | 0,11347891 |
| Mir301 | 0,02378357 | 0,69183824 | 0,1219867 |
| Mir224 | 0,02477974 | -0,84238681 | 0,12507868 |
| Mirlet7f-2 | 0,02920144 | -0,54877344 | 0,14509466 |
| Mir20a | 0,03063273 | 0,6358881 | 0,14986473 |
| Mir1668 | 0,031156 | -0,69232835 | 0,15011527 |
| Mir1933 | 0,03479776 | 1,09375916 | 0,1651595 |
| Mir30c-1 | 0,03597768 | -0,72756162 | 0,16617413 |
| Mir17 | 0,03605665 | 0,56433748 | 0,16617413 |
| Mir6395 | 0,03684046 | 1,11960249 | 0,16736094 |
| Mirlet7a-2 | 0,03761998 | -0,47925317 | 0,16849511 |
| Mir34a | 0,03903241 | -0,60997682 | 0,17158948 |
| Mir30c-2 | 0,03939004 | -0,6982525 | 0,17158948 |
| Mir7688 | 0,04047678 | 0,59903409 | 0,17394075 |
| Mir199b | 0,04227621 | -0,54086551 | 0,17925114 |
| Mir5122 | 0,04625776 | -0,64041146 | 0,19355222 |
| Mir5117 | 0,04983901 | 0,5427802 | 0,20582865 |
| Mir3069 | 0,05297039 | 1,06094393 | 0,21390645 |
| Mir7063 | 0,05314028 | 1,00418584 | 0,21390645 |
| Mir141 | 0,05686109 | -0,64704277 | 0,22602282 |
| Mir7044 | 0,06018267 | -0,84150998 | 0,23135054 |
| Mir32 | 0,06074255 | -0,66447846 | 0,23135054 |
| Mir125b-1 | 0,06080792 | -0,47568299 | 0,23135054 |
| Mir99b | 0,06266273 | -0,42535193 | 0,23135054 |
| Mir199a-2 | 0,06339928 | -0,48962913 | 0,23135054 |
| Mir199a-1 | 0,06343717 | -0,48963275 | 0,23135054 |
| Mir125b-2 | 0,06361491 | -0,46742523 | 0,23135054 |
| Mir6996 | 0,0643145 | 0,86866377 | 0,23135054 |
| Mir25 | 0,0652223 | 0,4077401 | 0,23135054 |
| Mir674 | 0,06578333 | -0,47056429 | 0,23135054 |
| Mir708 | 0,06620409 | -0,66358472 | 0,23135054 |
| Mir1947 | 0,07109368 | 0,51200918 | 0,24573685 |
| Mir5123 | 0,07317114 | 0,92106741 | 0,2501981 |
| Mir96 | 0,07459602 | -0,79000644 | 0,25235676 |
| Mir145b | 0,07699794 | 0,70556911 | 0,2566027 |
| Mir1247 | 0,07746496 | -0,89823418 | 0,2566027 |
| Mir429 | 0,08494198 | -0,65263314 | 0,27713725 |
| Mir6516 | 0,08540708 | 0,52793167 | 0,27713725 |
| Mir20b | 0,08780495 | 0,87015884 | 0,28204013 |
| Mir7224 | 0,08973457 | -0,6064725 | 0,28535592 |
| Mir672 | 0,09667695 | -0,73165346 | 0,3043888 |
| Mir133a-2 | 0,0989734 | 0,77070082 | 0,30856412 |
| Mir351 | 0,10265249 | -0,37081439 | 0,31692712 |
| Mir1199 | 0,10483176 | -0,85808084 | 0,32054325 |
| Mir329 | 0,11105211 | -0,84719287 | 0,33632925 |
| Mir297b | 0,11476248 | 0,78593539 | 0,34248711 |
| Mir1191 | 0,11523937 | 0,51255139 | 0,34248711 |
| Mir342 | 0,12496064 | -0,37197495 | 0,36793965 |
| Mir3086 | 0,12828404 | -0,68885109 | 0,37425986 |
| Mir188 | 0,13415924 | -0,51662084 | 0,38784218 |
| Mir6945 | 0,13549239 | 0,90195876 | 0,38816739 |
| Mir3098 | 0,13676639 | 0,80080296 | 0,38831885 |
| Mir344 | 0,14111815 | -0,89867439 | 0,39393898 |
| Mir3965 | 0,14122341 | 0,54763625 | 0,39393898 |
| Mir19b-1 | 0,14482497 | 0,49711413 | 0,39535027 |
| Mir297a-4 | 0,14563532 | 0,73257731 | 0,39535027 |
| Mir652 | 0,14687686 | 0,31296937 | 0,39535027 |
| Mir19b-2 | 0,14704408 | 0,49655174 | 0,39535027 |
| Mir7-2 | 0,14874534 | 0,68520277 | 0,39535027 |
| Mir350 | 0,14918878 | 0,41943552 | 0,39535027 |
| Mir34c | 0,15125821 | 0,55018184 | 0,39752158 |
| Mir7668 | 0,15438983 | -0,68585129 | 0,40242595 |
| Mir5114 | 0,1560603 | -0,45537925 | 0,40347297 |
| Mir879 | 0,15746568 | -0,55281242 | 0,40382329 |
| Mir7658 | 0,15933065 | 0,78569675 | 0,40533716 |
| Mirlet7b | 0,16154328 | -0,4536394 | 0,40770446 |
| Mir21a | 0,16678274 | 0,42705235 | 0,412005 |
| Mir193a | 0,16752006 | -0,53966646 | 0,412005 |
| Mirlet7c-2 | 0,16924806 | -0,37292083 | 0,412005 |
| Mir5129 | 0,16962411 | -0,59354804 | 0,412005 |
| Mir26b | 0,1702678 | -0,32735818 | 0,412005 |
| Mir365-2 | 0,17102094 | -0,38499037 | 0,412005 |
| Mir6540 | 0,17749792 | -0,53651313 | 0,42072487 |
| Mir365-1 | 0,17831952 | -0,38938317 | 0,42072487 |
| Mir1249 | 0,17860961 | -0,39633445 | 0,42072487 |
| Mir26a-2 | 0,18569481 | -0,30011038 | 0,43112073 |
| Mir26a-1 | 0,1857344 | -0,30007745 | 0,43112073 |
| Mirlet7d | 0,19076919 | -0,2822353 | 0,43800365 |
| Mir3088 | 0,19145443 | 0,69613162 | 0,43800365 |
| Mir344-2 | 0,19498395 | -0,77590047 | 0,44289212 |
| Mir1955 | 0,19742847 | 0,6498802 | 0,44422879 |
| Mir5116 | 0,19836631 | 0,71258181 | 0,44422879 |
| Mir214 | 0,20616945 | -0,41865206 | 0,45847472 |
| Mir6899 | 0,21940026 | 0,3753005 | 0,48450891 |
| Mir190b | 0,22094098 | 0,43151704 | 0,48454643 |
| Mir151 | 0,22315708 | -0,29314859 | 0,48605447 |
| Mir6980 | 0,22571649 | -0,4584442 | 0,48828466 |
| Mir30d | 0,22811517 | -0,24660558 | 0,49013936 |
| Mir6952 | 0,23388116 | 0,52986981 | 0,49850566 |
| Mir7035 | 0,23514418 | 0,63159061 | 0,49850566 |
| Mir7015 | 0,25185285 | 0,39545313 | 0,53000886 |
| Mir215 | 0,25333757 | 0,41831865 | 0,53000886 |
| Mir7049 | 0,25585227 | -0,58308964 | 0,53177138 |
| Mir449a | 0,25978739 | -0,38814155 | 0,53567672 |
| Mir3475 | 0,26110029 | -0,68511559 | 0,53567672 |
| Mir3963 | 0,27053978 | 0,47404108 | 0,55148494 |
| Mir33 | 0,28587516 | 0,29332158 | 0,57891102 |
| Mir3572 | 0,28763503 | 0,49510727 | 0,57891102 |
| Mir1941 | 0,29218836 | -0,4074045 | 0,58437672 |
| Mir152 | 0,29721416 | -0,26734089 | 0,58789737 |
| Mir1948 | 0,29764615 | 0,57498078 | 0,58789737 |
| Mirlet7g | 0,30524888 | -0,21681715 | 0,59372832 |
| Mir195b | 0,30605081 | -0,60817457 | 0,59372832 |
| Mir27a | 0,30758596 | -0,24325075 | 0,59372832 |
| Mir484 | 0,30806658 | -0,21388383 | 0,59372832 |
| Mir181b-2 | 0,31780319 | -0,22124858 | 0,60880371 |
| Mir3057 | 0,3227448 | 0,30558892 | 0,61348677 |
| Mir671 | 0,32540628 | -0,27942416 | 0,61348677 |
| Mir181b-1 | 0,32603542 | -0,21673234 | 0,61348677 |
| Mir702 | 0,32885874 | -0,35679782 | 0,61515929 |
| Mir6985 | 0,33264538 | -0,49562969 | 0,61794958 |
| Mir700 | 0,33423688 | -0,3558073 | 0,61794958 |
| Mir23a | 0,33863276 | -0,22903533 | 0,62172839 |
| Mir872 | 0,340191 | -0,22310609 | 0,62172839 |
| Mir541 | 0,34795888 | -0,28814572 | 0,6322307 |
| Mir184 | 0,35091507 | -0,39997821 | 0,6322307 |
| Mir1264 | 0,35373658 | -0,29207966 | 0,6322307 |
| Mir187 | 0,35569359 | -0,29231957 | 0,6322307 |
| Mir130a | 0,35601145 | -0,20092078 | 0,6322307 |
| Mir1968 | 0,35786643 | -0,28270026 | 0,6322307 |
| Mir8103 | 0,36401345 | 0,2737296 | 0,6368489 |
| Mir192 | 0,36448585 | 0,21180471 | 0,6368489 |
| Mir410 | 0,37525224 | -0,26810816 | 0,65207767 |
| Mir3962 | 0,38412681 | -0,23781122 | 0,66098563 |
| Mir3102 | 0,38886485 | -0,36206625 | 0,66098563 |
| Mir744 | 0,38961904 | 0,21003082 | 0,66098563 |
| Mir326 | 0,393184 | -0,23824948 | 0,66098563 |
| Mir153 | 0,39336565 | -0,3573597 | 0,66098563 |
| Mir6943 | 0,39348266 | 0,37109604 | 0,66098563 |
| Mir7046 | 0,39548812 | -0,27826507 | 0,66098563 |
| Mir6979 | 0,39700709 | -0,50267626 | 0,66098563 |
| Mir7656 | 0,40011872 | 0,3754614 | 0,66269663 |
| Mir378b | 0,40680857 | -0,32644292 | 0,67028562 |
| Mir5620 | 0,420692 | -0,45538507 | 0,68842366 |
| Mir1983 | 0,42214658 | 0,29749075 | 0,68842366 |
| Mir210 | 0,42713911 | 0,21842708 | 0,69301141 |
| Mir21c | 0,43264374 | 0,37489865 | 0,69837923 |
| Mir3109 | 0,43878232 | -0,33034806 | 0,704711 |
| Mir6946 | 0,44728409 | -0,34785172 | 0,71160943 |
| Mir1930 | 0,4475531 | 0,41208674 | 0,71160943 |
| Mir3097 | 0,45983379 | -0,27911476 | 0,72749824 |
| Mir7036b | 0,46376903 | 0,41831666 | 0,72954979 |
| Mir34b | 0,46663065 | 0,23155973 | 0,72954979 |
| Mir221 | 0,46816164 | 0,16146107 | 0,72954979 |
| Mir511 | 0,47271038 | 0,26352386 | 0,72954979 |
| Mir205 | 0,47361112 | 0,34608494 | 0,72954979 |
| Mir1912 | 0,47597234 | -0,27961285 | 0,72954979 |
| Mir186 | 0,47847997 | 0,14907323 | 0,72954979 |
| Mir1981 | 0,47948398 | -0,17782888 | 0,72954979 |
| Mir8094 | 0,48472925 | 0,23948678 | 0,73401858 |
| Mir7k | 0,48715701 | 0,36095777 | 0,73419872 |
| Mir542 | 0,49261263 | -0,18834501 | 0,73454871 |
| Mir676 | 0,4944791 | -0,21835947 | 0,73454871 |
| Mir101c | 0,49575769 | -0,16560373 | 0,73454871 |
| Mir551b | 0,49662884 | -0,22097816 | 0,73454871 |
| Mir29a | 0,50085801 | -0,14134298 | 0,73583969 |
| Mir322 | 0,50463891 | -0,14768303 | 0,73583969 |
| Mir7213 | 0,50588741 | -0,22291253 | 0,73583969 |
| Mir682 | 0,50675753 | 0,34867568 | 0,73583969 |
| Mir496a | 0,51389158 | -0,3465293 | 0,74246088 |
| Mir1843a | 0,51598696 | 0,14289037 | 0,74246088 |
| Mir328 | 0,51978915 | 0,16534106 | 0,74332787 |
| Mir3068 | 0,52213843 | 0,34285918 | 0,74332787 |
| Mir1964 | 0,5272703 | 0,25243716 | 0,74332787 |
| Mir203 | 0,52729828 | -0,1919632 | 0,74332787 |
| Mir3081 | 0,52827704 | -0,42203631 | 0,74332787 |
| Mir874 | 0,54000461 | -0,18197095 | 0,75144368 |
| Mir425 | 0,54133276 | 0,14999847 | 0,75144368 |
| Mir10a | 0,54252047 | -0,20394138 | 0,75144368 |
| Mir490 | 0,54514669 | -0,16143901 | 0,75144368 |
| Mir3060 | 0,54586003 | -0,18869458 | 0,75144368 |
| Mir1943 | 0,54857905 | 0,17990373 | 0,75193163 |
| Mir28c | 0,5514671 | -0,15214135 | 0,75264609 |
| Mir450b | 0,55479074 | 0,17551928 | 0,75394639 |
| Mir501 | 0,55746594 | -0,14208609 | 0,75418713 |
| Mir1839 | 0,55971121 | 0,14741966 | 0,75418713 |
| Mir138-1 | 0,57505142 | -0,21095392 | 0,77158798 |
| Mir331 | 0,58382675 | -0,19616128 | 0,77719924 |
| Mir9-3 | 0,58412144 | -0,32936549 | 0,77719924 |
| Mir6975 | 0,60095094 | -0,30249973 | 0,79626 |
| Mir194-2 | 0,60373351 | -0,13794807 | 0,79660906 |
| Mir7219 | 0,60694615 | -0,19594359 | 0,79660906 |
| Mir324 | 0,60872957 | -0,14825001 | 0,79660906 |
| Mir211 | 0,62301445 | -0,25720472 | 0,81196146 |
| Mir29c | 0,63307586 | -0,11414204 | 0,82170663 |
| Mir6538 | 0,63602619 | -0,20584593 | 0,8221802 |
| Mir7010 | 0,6437495 | -0,20682076 | 0,8287949 |
| Mir1961 | 0,64948776 | -0,24722534 | 0,83281092 |
| Mir449c | 0,65372617 | 0,18450029 | 0,8348792 |
| Mir3103 | 0,66098523 | 0,21879753 | 0,84027613 |
| Mir3473d | 0,66323682 | -0,14475948 | 0,84027613 |
| Mir6988 | 0,66829951 | -0,18431585 | 0,84057508 |
| Mir7019 | 0,66875942 | -0,19465438 | 0,84057508 |
| Mir5130 | 0,68183359 | 0,23702983 | 0,84561222 |
| Mir5107 | 0,68204073 | 0,2494473 | 0,84561222 |
| Mir5131 | 0,68396246 | 0,16460935 | 0,84561222 |
| Mir194-1 | 0,68712139 | -0,11216121 | 0,84561222 |
| Mir703 | 0,6875613 | -0,25797903 | 0,84561222 |
| Mir6914 | 0,68904454 | -0,18975608 | 0,84561222 |
| Mir6911 | 0,69138106 | 0,19186589 | 0,84561222 |
| Mir3082 | 0,70910808 | 0,11161343 | 0,86397076 |
| Mir455 | 0,71549341 | -0,1543354 | 0,865845 |
| Mir434 | 0,71609193 | -0,09714814 | 0,865845 |
| Mir6418 | 0,7212435 | -0,1581356 | 0,86877057 |
| Mir212 | 0,74366878 | 0,11913012 | 0,89240254 |
| Mir6989 | 0,74659901 | 0,11106611 | 0,89255069 |
| Mir103-2 | 0,75142676 | 0,06596055 | 0,89495771 |
| Mir103-1 | 0,75841435 | 0,064084 | 0,89990956 |
| Mir7031 | 0,76336882 | 0,19463942 | 0,90242113 |
| Mir7075 | 0,76688445 | 0,11644408 | 0,90321946 |
| Mir877 | 0,77516325 | -0,08698604 | 0,90960116 |
| Mir3964 | 0,77806935 | 0,13758729 | 0,90965461 |
| Mir667 | 0,78347096 | 0,14988454 | 0,91137629 |
| Mir7083 | 0,78785622 | -0,08487906 | 0,91137629 |
| Mir7052 | 0,78813987 | -0,12630095 | 0,91137629 |
| Mir345 | 0,79765514 | -0,08169505 | 0,91458727 |
| Mir8112 | 0,79865867 | -0,06631225 | 0,91458727 |
| Mir22 | 0,80001205 | -0,06861952 | 0,91458727 |
| Mir362 | 0,80242091 | -0,08380609 | 0,91458727 |
| Mir7043 | 0,8103585 | -0,12271898 | 0,92033573 |
| Mir320 | 0,81989765 | 0,0518627 | 0,92513038 |
| Mir1843b | 0,82251512 | 0,05077025 | 0,92513038 |
| Mir7j | 0,82871382 | -0,06267842 | 0,92513038 |
| Mir448 | 0,83514302 | -0,07179212 | 0,92513038 |
| Mir222 | 0,83549136 | -0,05077577 | 0,92513038 |
| Mir7-1 | 0,83639831 | -0,06592726 | 0,92513038 |
| Mir378d | 0,83643339 | 0,08759031 | 0,92513038 |
| Mir181a-1 | 0,83985884 | -0,0480991 | 0,92513038 |
| Mir181a-2 | 0,84076314 | -0,0478173 | 0,92513038 |
| Mir673 | 0,86313888 | -0,06181261 | 0,93713965 |
| Mir1191b | 0,86337898 | -0,05963968 | 0,93713965 |
| Mir107 | 0,86521821 | -0,0367633 | 0,93713965 |
| Mir101a | 0,86980911 | -0,03490147 | 0,93713965 |
| Mir1966 | 0,87089739 | -0,06305869 | 0,93713965 |
| Mir339 | 0,87149967 | -0,03862794 | 0,93713965 |
| Mir378c | 0,87230609 | -0,05540216 | 0,93713965 |
| Mir181d | 0,87543316 | -0,03640128 | 0,93733248 |
| Mir532 | 0,88391681 | 0,03523278 | 0,94018136 |
| Mir15a | 0,88400701 | 0,0385791 | 0,94018136 |
| Mir16-1 | 0,88930528 | 0,02982422 | 0,94266359 |
| Mir7068 | 0,90000654 | -0,04119561 | 0,94567678 |
| Mir16-2 | 0,90005293 | 0,02693141 | 0,94567678 |
| Mir101b | 0,90247836 | 0,0280985 | 0,94567678 |
| Mir361 | 0,90404322 | -0,02663024 | 0,94567678 |
| Mir1982 | 0,91637065 | 0,04269859 | 0,95542907 |
| Mir28a | 0,92212628 | -0,02105646 | 0,9582881 |
| Mir146 | 0,93335809 | 0,02766513 | 0,96680089 |
| Mir450-1 | 0,93776067 | -0,01875632 | 0,96776139 |
| Mir8111 | 0,9405163 | -0,0302403 | 0,96776139 |
| Mir5106 | 0,94446562 | -0,05104298 | 0,96776139 |
| Mir3061 | 0,94645847 | 0,02152821 | 0,96776139 |
| Mir7235 | 0,95240256 | 0,02594993 | 0,96985401 |
| Mir450-2 | 0,95460473 | -0,01368172 | 0,96985401 |
| Mir433 | 0,96837728 | 0,02057595 | 0,9807133 |
| Mir1298 | 0,97155238 | -0,01106237 | 0,98080526 |
| Mir8114 | 0,98094094 | 0,00670874 | 0,98714943 |
| Mir764 | 0,99429735 | 0,00228359 | 0,99743393 |
| Mir431 | 0,99902189 | 0,00037598 | 0,99902189 |

**Supplemental Table 3.** Organ parameters of endotoxemic mice

|  | **GOT** | **GPT** | **Glucose** | **Creatinine** | **Urea** | **LDH** |
| --- | --- | --- | --- | --- | --- | --- |
| **vehicle CTR** | 65.9 ±15.8 | 42.1 ±43.0 | 11.6 ±1.3 | 55.7 ±6.9 | 9.5 ±2.5 | 187.1 ±99.4 |
| **LPS MIR155 +/+** | 264.0 ±85.4 | 107.5 ±41.9 | 7.6 ±1.9 | 50.7 ±14.9 | 26.1 ±6.5 | 930.1 ±149.3 |
| **LPS MIR155 +/-** | 192.3 ±49.1 | 225,7 ±164.9 | 8.2 ±2.2 | 37.5 ±2.2 | 26.2 ±9.6 | 723.5 ±485.6 |
| **LPS MIR155 -/-** | 311.0 ±350.4 | 303.9 ±534.6 | 6,6 ±2.8 | 45.7 ±9.7 | 26.6 ±8.3 | 833.5 ±416.2 |

Mice were challenged with either 17.5mg/kg BW LPS (n=10-21) or vehicle (0.9% NaCl, n=8) and sacrificed after 16 hours. Organ parameters from serum and urine were measured and standard deviation was calculated. The heterozygous MIR155^+/-^ mice seem to have a slightly better organ function compared to the homozygous MIR155^+/+^ and MIR155^-/-^ mice.

**Supplemental Table 4.** Activity score to evaluate severity of illness in septic mice.

| **Score** | **Activity** | **General Condition** | **Behavior and Actions** |
| --- | --- | --- | --- |
| **1** | Very active | Smooth fur, clear clean eyes, clean orifices | Vigilant, curious, regular movements |
| **2** | Active | Smooth fur, clear clean eyes, clean orifices | Vigilant, normal movements |
| **3** | Less active | Dull fur, eyes not fully open | Vigilant, quiet, weakened movements, normal posture, neglected body care |
| **4** | Restricted | Dull fur, standing fur, eyes not fully open, unkempt orifices | Quiet, frequent persistence, restricted reaction on environmental stimuli, neglected body care |
| **5** | Apathetic | Dirty, dull fur, eyes closed, clogged orifices, stooped posture | Self-isolation, no significant activity |
| **6** | Moribund  (Death is expected) | Eyes closed, lateral position, shallow breathing, cramps, reduced body temperature | No activity, no reaction on environmental stimuli |
